# PROMOTING AFHS FRAMEWORK WITHIN NURSING STAFF TRAINING CURRICULUM: NURSING HOME QUALITY IMPROVEMENT INITIATIVE

**DOI:** 10.1093/geroni/igad104.2615

**Published:** 2023-12-21

**Authors:** Oksana Shnayder, Sweta Tewary, Naushira Pandya

**Affiliations:** Nova Southeastern University, Davie, Florida, United States; Nova Southeastern University, Davie, Florida, United States; Nova Southeastern University, Kiran Patel College of Osteopathic Medicine, Davie, Florida, United States

## Abstract

The recent report by the Committee on the Quality of Care in Nursing Homes highlighted the limitations within nursing home facilities including neglect and abuse of their residents, high living costs and poor quality of life, lack of oversight, as well as nursing staff shortages and burnouts. With multiple challenges in sustaining the nursing home workforce, it is imperative to provide support and continued education to nursing home staff and nursing students on recent developments in evidence-based approaches to geriatric care including Age-Friendly Health Systems (AFHS) and dementia-friendly care. NSU SFGWEP conceptualized, designed, and delivered a modified geriatric curriculum to the nursing workforce at three distinct partner sites. The curriculum integrated eight training modules within the framework of AFHS and covers topics on each of the 4Ms: Matters Most, Mobility, Mentation, and Medication. The trainings are currently being delivered to the nursing home staff, nursing students, and to CNA students. The data on increase in education on AFHS and the 4Ms is being collected via a specifically designed survey which is administered electronically through mobile devices or via paper questionnaires. The survey responses are managed through REDCapTM, a secure web application for administering online surveys. An estimated 160 trainees have completed at least one training session and 38 trainees have submitted the feedback survey. Preliminary data on pre/post-training evaluations suggests an improvement in participants’ knowledge of the geriatric topic within AFHS and the 4Ms, proposing a sustainable training program for the nursing workforce to promote age-friendly care for older adults

